# Efficacy of Different Materials for Maxillary Sinus Floor Augmentation With Lateral Approach. A Systematic Review

**DOI:** 10.1111/cid.70053

**Published:** 2025-05-22

**Authors:** Massimo Del Fabbro, Silvio Taschieri, Stefano Corbella

**Affiliations:** ^1^ Department of Biomedical, Surgical and Dental Sciences UniversitÀ Degli Studi Di Milano Milan Italy; ^2^ Fondazione IRCCS CA’ Granda Ospedale Maggiore Policlinico Milan Italy; ^3^ Dental Clinic, IRCCS Galeazzi Sant'Ambrogio Hospital Milan Italy

**Keywords:** bone graft, bone substitutes, dental implants, graft stability, implant survival, maxillary sinus floor augmentation

## Abstract

**Background:**

Maxillary sinus floor augmentation (MSFA) with lateral approach has undergone considerable changes since its inception, mainly due to the introduction of a variety of grafting materials and surgical protocols, with the aim of providing predictable and durable support to implants and improving treatment outcomes. The aim of this systematic review was to evaluate the performance of MSFA in terms of graft stability and implant survival, based on randomized clinical trials.

**Methods:**

The study protocol was registered on PROSPERO. An electronic search was performed on Medline, Embase, and CENTRAL databases, integrated with hand‐searching on the main pertinent Journals and search of gray literature. Randomized studies on MSFA with a lateral approach reporting on radiographic changes in graft height or volume after at least six months of healing, implant survival, and marginal bone level changes after at least 12 months of loading were included. Two independent reviewers selected the studies. Studies included underwent data extraction and risk of bias assessment using the ROB 2.0 Cochrane tool.

**Results:**

Out of 3922 studies retrieved, 49 studies (57 articles) were included for data extraction and qualitative analysis. These studies reported on 1265 patients and 1722 MSFA procedures. Thirteen studies were at low risk, 30 had some concerns, and six were at high risk of bias. No quantitative synthesis was possible due to the characteristics of the studies and their methodology. The overall implant survival rate ranged from 84.6% to 100% without evidence of any relevant difference related to the grafting material.

**Conclusion:**

The results of this review, based on descriptive statistics, may suggest that the success of the lateral MSFA procedure is independent of the graft type, at least in the short term. To verify this interpretation, formal statistical analyses on trustworthy and more detailed clinical data are needed. Also, long‐term data on graft dimensional stability from evidence‐based studies are needed.

## 1 Introduction

Maxillary sinus floor augmentation (MSFA) is a popular surgical procedure used to increase the bone volume in the atrophic posterior upper jaw, to facilitate dental implant placement and subsequent rehabilitation of mastication function. This technique, introduced in the early 80s by Boyne and Tatum [1, 2], is commonly performed in cases where the patient lacks sufficient bone height in the posterior maxilla, mainly due to sinus pneumatization and alveolar bone resorption following tooth loss [3, 4, 5].

Various types of grafting materials and combinations are utilized for sinus augmentation procedures, each with its own set of advantages and disadvantages [6, 7, 8]. Implant placement may be performed in the same surgical session as grafting, when the residual bone is sufficient to grant adequate implant stability, or in a delayed procedure, leaving at least 4–6 months to allow for graft maturation and consolidation [9]. Graftless techniques have also been proposed, consisting of lifting the Schneiderian membrane before placing the implant and using the latter to keep the membrane elevated with respect to the sinus floor (the so‐called tent effect) [10, 11].

MSFA can be performed using a lateral or a transalveolar (crestal) approach [3]. The lateral approach is generally indicated for more severe atrophies; an osteotomy is performed to create a window in the lateral sinus wall through which to visualize the surgical site and perform membrane elevation and grafting procedure [5]. The transalveolar technique involves the use of specific osteotomes to condense the bone crestally, create the space for positioning the implant, and elevate the sinus floor. The latter technique is more conservative than the lateral approach, avoiding sinus wall osteotomy, and is generally indicated for moderate atrophies [4]. As any bone regeneration procedure, MSFA may be associated with several complications, impacting the outcomes of the treatment [12, 13]. Therefore, less invasive procedures may be considered for the treatment of maxillary posterior atrophy, such as the placement of short (or very short) dental implants [14].

Previous systematic reviews investigated the effectiveness of the MSFA elevation procedure considering the clinical and histomorphometric outcomes [6, 15, 16, 17]. In general, as it appears from the study on histomorphometric analysis, there is no evidence of superior outcomes of any bone substitute material over the others [18]. However, there is a lack of information in the literature about which combination of biomaterials or which technique or surgical approach may lead to higher bone volume formation or to better clinical outcomes as measured in the short term.

For such reason, this systematic review aimed to investigate if the choice of a grafting material, a combination of materials, or of different surgical approaches may have an effect on the implant survival rate, or the stability of the augmented bone in the MSFE with lateral approach, based only on randomized clinical studies.

## 2 Materials and Methods

The protocol of the present systematic review of the literature was registered in PROSPERO before the beginning of the screening of papers for inclusion (registration number CRD42024597602). The manuscript was prepared following the checklist of the PRISMA (Preferred Reporting Items for Systematic reviews and Meta‐Analyses) statement [19], and the review was carried on following the guidelines of the Cochrane Handbook for Systematic Review of Interventions—Second Edition [20].

The main purpose of the present systematic review of the literature is to answer the following focused questions:

– Focused Question 1 (FQ1): in patients requiring MSFA with a lateral approach, what is the efficacy, in terms of the size and stability of bone augmentation, of different techniques and grafting materials based on the analysis of randomized controlled clinical trials (RCTs) with at least a 6‐month follow‐up from surgical intervention?
– Focused Question 2 (FQ2): in patients receiving dental implants after maxillary sinus floor augmentation (MSFA) with lateral approach what is the efficacy, in terms of implant survival, success, and peri‐implant bone resorption, of different techniques and grafting materials, based on the analysis of RCTs with at least one year follow‐up from prosthetic loading?

### 2.1 Eligibility Criteria

Based on the PICOS, the criteria for considering the studies for inclusion were the following.

#### 2.1.1 Population (P)

For FQ1 and FQ2, patients requiring MSFA with a lateral approach procedure with any size of residual alveolar bone height in latero‐posterior areas of the maxilla.

#### 2.1.2 Intervention (I)

For FQ1 and FQ2, MSFA with lateral approach with any technique and any combination of grafting materials.

#### 2.1.3 Control (C)

For FQ1 and FQ2, MSFA with a different technique or combination of grafting materials.

#### 2.1.4 Outcomes (O)

For FQ1, the primary outcomes were the size and the stability of bone augmentation, as measured through radiographic examination (either bidimensional or tridimensional or both), by comparing presurgical and postsurgical documentation at different follow‐up periods.

For FQ2, the primary outcomes were the implant success, as defined by standard criteria, and survival rates of implants placed in augmented bone after MSFA with lateral approach and peri‐implant bone resorption rate over time after prosthetic loading.

For FQ1 and FQ2, the secondary outcomes were patient‐reported outcome measures (PROMs) and the occurrence of complications/adverse events.

#### 2.1.5 Studies (S)

For FQ1, RCTs presenting outcomes measured at least six months from surgical intervention after MSFA with lateral approach intervention.

For FQ2, RCTs with at least one‐year follow‐up after prosthetic loading.

### 2.2 Search Strategy and Sources

The following electronic sources were searched for articles to be potentially included in the review: MEDLINE through Ovid interface, EMBASE, and Cochrane CENTRAL. The search strategy that was adopted is presented in Appendix 1. Also, gray literature was searched by using Greylit and OpenGrey search engines. The authors of the review also screened trials registries for pertinent research (ClinicalTrials.gov and EU Clinical Trials Register).

Moreover, a manual search was performed by screening the reference lists of the included papers and all the issues published since 2000 of the following journals: *Clinical Implant Dentistry and Related Research*, *Clinical Oral Investigations*, *Clinical Oral Implants Research*, *Journal of Clinical Periodontology*, *Journal of Periodontology*, *Journal of Dentistry*, *Journal of Dental Research*, *Implant Dentistry*, *International Journal of Oral and Maxillofacial Implants*, *International Journal of Oral Implantology*, *International Journal of Periodontics and Restorative Dentistry*, and *Journal of Oral Implantology*.

The last electronic search was performed on May 29, 2024.

### 2.3 Selection Process

The following information was retrieved from included papers and recorded by two authors (Massimo Del Fabbro, Stefano Corbella): authors' names, year of publication, country, setting, study type (parallel or split‐mouth RCT), number of subjects per group, number and characteristics (brand, surface type) of implants placed per group, characteristics of the population, baseline radiographic characteristics (i.e., residual ridge height at the intended implant site), description of the techniques, grafting materials, follow‐up duration, radiographic, clinical, and patient‐reported outcomes at any follow‐up, adverse events (e.g., implant failures and the time they occurred), and complications (type and time they occurred). In case of missing information, the authors of the papers were contacted by email up to twice for providing missing data.

### 2.4 Risk of Bias and Quality of Evidence Assessment

The risk of bias was evaluated through the RoB 2.0 tool for RCTs [20]. A study was judged at high risk of bias if at least one of the domains presented high risk of bias or if more than two domains presented some concerns about risk of bias; the study was considered at low risk if all domains were at low risk of bias; in any other cases, the study was judged to have some concerns about risk of bias. The quality of evidence was evaluated by using the GRADE framework [21].

### 2.5 Data Synthesis and Analysis

It was planned to perform a meta‐analysis for each outcome considered by grouping the studies based on the technique used, on the grafting materials, and on the follow‐up time. However, due to the characteristics of the included studies and also because of the presence of unit‐of‐analysis errors in several studies, no attempt to perform a meta‐analysis or summary statistics was provided. A qualitative synthesis of the studies included and of their results is presented, considering different bone grafting materials and techniques.

## 3 Results

**FIGURE 1:**
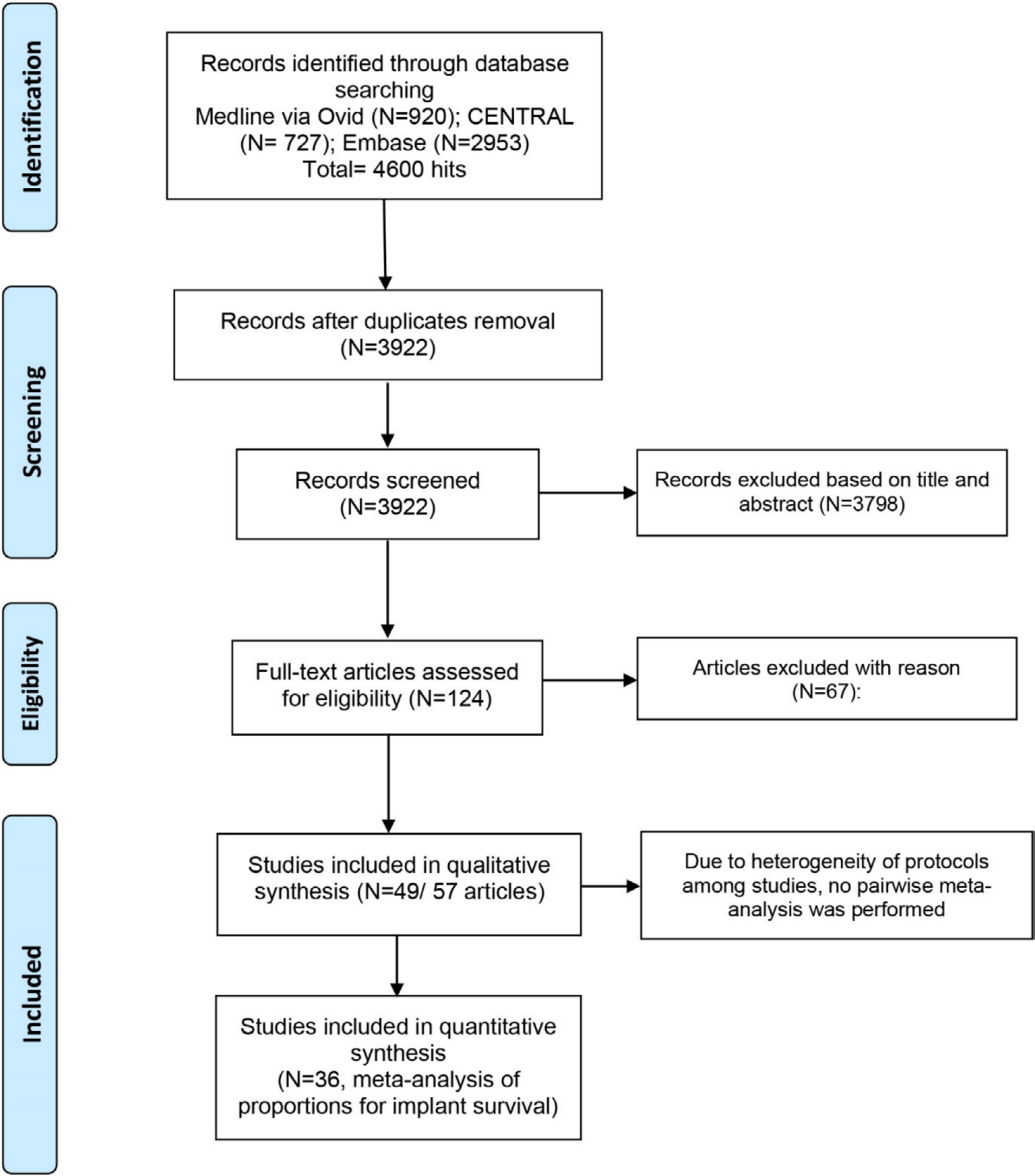
Flowchart of the selection process.

The article selection process is summarized in Figure 1. The general characteristics of the included studies are presented in Table 1. A total of 49 randomized studies (57 articles) [8, 22, 23, 24, 25, 26, 27, 28, 29, 30, 31, 32, 33, 34, 35, 36, 37, 38, 39, 40, 41, 42, 43, 44, 45, 46, 47, 48, 49, 50, 51, 52, 53, 54, 55, 56, 57, 58, 59, 60, 61, 62, 63, 64, 65, 66, 67, 68, 69, 70, 71, 72, 73, 74, 75, 76, 77] met the inclusion criteria. Six studies were reported in multiple publications [25, 26, 27, 31, 32, 33, 35, 36, 40, 41, 68, 69]. Overall, the included studies involved 1265 patients and 1722 surgical procedures for maxillary sinus augmentation. The studies excluded after full‐text analysis and the main reason for exclusion are listed in Appendix 2.

### 3.1 Risk of Bias Evaluation

The risk of bias evaluation, whose results are (Table 1) presented in Table 2, revealed that 13 studies were considered at low risk of bias, some concerns about risk of bias were raised in 30 studies, and 6 studies were at high risk (due to the bias arising from randomization/allocation process and to the characteristics of the sample). The main issues that caused the judgment regarded the randomization process and the blinding of the operators/patients. The risk of bias also considered (potential selection bias) when incorrect/inappropriate statistical analysis was performed.

**TABLE 1:** Main characteristics of the included studies.

Study	RCT type	Total patients treated	Total sinuses treated	Total implants placed	Simultaneous/delayed placement	FQ1 (graft size) FQ2 (survival/MBL)	Follow‐up FQ1/FQ2*	Test group(s): grafting material/surgical procedure	Control group(s): grafting material/surgical procedure
Adali et al. [22]	Split‐mouth	10	20	52	Delayed	FQ1/FQ2	6 m/12 m	Allograft + CGF	Allograft
Aldahouk et al. [23]	Mixed	20	30	38	Simultaneous	FQ1	6 m/—	Graftless 5 mm entry antrostomy	Graftless 10 mm entry antrostomy
Amam et al. [24]	Split‐mouth	9	18	NR	Delayed	FQ1	6 m/—	A‐PRF + β‐TCP	A‐PRF + CS (calcium sulfate)
Attia et al. [25], Schaaf et al. [26, 27]	Mixed	37	NR	210	Delayed	FQ2	–/11.3–15.1y (T: 12.53y, C: 12.90y)	ABG + PRP	ABG
Baldini et al. [28]	Split‐mouth	16	32	67	Delayed	FQ1	6 m/—	Small access window (6 × 6 mm) + DBBM Pen	Large access window (10 × 8 mm) + DBBM 1–2 mm
Chang et al. [29]	Parallel	54	54	84	Simultaneous/delayed	FQ1 FQ2	6 m/12 m	DBBM + gelatin sponge	DBBM
Cinar et al. [30]	Parallel	20	20	39	Delayed	FQ1	6 m/—	MPM: β ‐TCP + PRF	β‐TCP
Correia et al. [31, 32, 33]	Split‐mouth	12	24	38	Simultaneous	FQ1/FQ2	6 m/36 m	DPBM collagenated (Osteobiol mp3)	Intraoral ABG
da Silva et al. [34]	Split‐mouth	13	26	33	Delayed	FQ1/FQ2	6 m/36 m	Lumina‐bone porous (anorganic bovine bone)	DBBM
de Almeida Malzoni et al. [35], Pichotano et al. [36]	Mixed (3 groups)	24	36	46	Delayed	FQ1/FQ2	4 m; 8 m/12 m	L‐PRF + DBBM_ 4 m (T4), L‐PRF + DBBM_ 8 m (T8)	DBBM (0.25–1 mm)
Felice et al. [37]	Parallel	60	60	135	Delayed	FQ2	–/12 m	DBBM (1‐stage)	DBBM (2‐stage)
Galindo‐Moreno et al. [38]	Split‐mouth	10	20	NR	Delayed	FQ1	6 m/—	DBBM + ABG 80:20	DPBM + ABG 80:20
Galindo‐Moreno et al. [39]	Split‐mouth	8	16	NR	Delayed	FQ1	6 m/—	Biphasic phycogenic biomaterial + ABG 80:20	DBBM + ABG 80:20
Gorla et al. [40], dos Santos Pereira et al. [41]	Mixed	22	32	NR	Delayed	FQ1	6 m/—	T1 ABG + β‐TCP 1:1, T2 β ‐TCP	ABG
Hirota et al. [42]	Parallel	20	20	NR	Simultaneous	FQ1	9 m/—	DPBM + membrane subjacent to sinus mucosa	DPBM
Jia et al. [43]	Parallel	26	26	38	Delayed	FQ1/FQ2	12 m/12 m	DBBM + bone window	DBBM
Kaarthikeyan et al. [44]	Split‐mouth	7	14	NR	Delayed	FQ1	12 m/—	Blood clot (graftless)	PRF
Kamolratanakul et al. [45]	Parallel	32	32	32	Delayed	FQ2	–/12 m	DBBM (1–2 mm)	DBBM (0.25–1 mm)
Kandel et al. [46]	Parallel	14	18	23	Delayed	FQ1	6 m/—	Absorbable gelatin sponge	ABBM
Kawakami et al. [47]	Parallel	20	20	NR	Simultaneous	FQ1	9 m/—	Group A: antrostomy close to the level of the sinus floor; OsteoBiol GenOs	Group B: antrostomy 3–4 mm cranially to sinus floor; OsteoBiol GenOs
Khaled et al. [48]	Parallel	19	20	50	Delayed	FQ1	6 m/—	NHA	Graftless
Kiliç and Güngörmüş [49]	Parallel	18	18	NR	Delayed	FQ1	6 m/—	β‐TCP + PRP	β‐TCP
Kim et al. [50]	Parallel	42	46	NR	Delayed	FQ1	6 m/—	rhBMP‐2 + BCP	DBBM
Krennmair et al. [51]	Mixed	28	30	56	Delayed	FQ1/FQ2	6 m; 24 m/12 m; 36 m	DPBM (0.25–1 mm)	DBBM (0.25–1 mm)
Kühl et al. [52]	Split‐mouth	8	16	NR	Simultaneous	FQ1	6 m/—	BCP (HA + β ‐TCP 60:40) + ABG 1:1	BCP alone
Kühl et al. [53]	Split‐mouth	13	26	NR	Delayed	FQ1	6 m/—	DBBM + tibial BMA; DBBM + iliac BMAC	DBBM‐tibial BMA; DBBM‐iliac BMAC
Lee et al. [54]	Mixed	16	20	NR	Delayed	FQ1	6 m/—	DPBM	DBBM
Lie et al. [55]	Split‐mouth	10	20	59	Delayed	FQ1/FQ2	4 m/57–88 m	Graftless (lift with resorbable membrane)	ABG + xenograft
Lindgren et al. [56]	Split‐mouth	11	22	62	Delayed	FQ2	–/12/36 m	BCP	DBBM
Liu et al. [57]	Parallel	19	20	27	Simultaneous	FQ1	5 m; 8 m/—	DBBM 5 m	DBBM 8 m
Meloni et al. [58]	Parallel	16	16	32	Delayed	FQ2	–/12 m	DBBM + ABG 1:1	DBBM 100%
Menezes et al. [59]	Parallel	40	40	NR	Delayed	FQ1	6 m/—	G2: β –TCP; G3: β‐TCP + ABG 1:1; G4: bioact glass; G5: bioglass + ABG 1:1	G1: ABG
Nizam et al. [60]	Split‐mouth	13	26	58	Delayed	FQ1/FQ2	6 m/12 m	DBBM (DBBM) + L‐PRF	DBBM
Pang et al. [61]	Mixed	25	28	NR	Delayed	FQ1	6 m/—	Calcium phosphate double‐coated anorganic bovine bone (InduCera)	DBBM
Pereira et al. [62]	Mixed	29	35	NR	Simultaneous	FQ1	6 m/—	Bioactive glass (Biogran) Bioactive glass + ABG 1:1	ABG
Scarano et al. [63]	Mixed	23	28	NR	Delayed	FQ1	6 m/—	Collagenated porcine bone (Geno‐os)	Heterologous cortical lamina (OsteoBiol)
Sedeqi et al. [64]	Mixed	16	20	26	Simultaneous	FQ1	6 m/—	ABBM	ABBM + CA (0.8:1)
Sokolowski et al. [65]	Mixed	20	32	51	Delayed	FQ1	6 m; 12 m; 24 m/—	BCP	Almost pure HA
Stacchi et al. [66]	Split‐mouth	28	56	107	Delayed	FQ2	–/12 m	Pure sintered NHA (Fisiograft, Ghimas)	DBBM
Starch‐Jensen et al. [67]	Parallel	60	60	60	Delayed	FQ2	–/12 m	TGI) 1:1 ABG and APBM; TGII) 1:1 ABG and BBGM	ABG (2 cc) from the zygomatic buttress
Starch‐Jensen et al. [68]	Parallel	40	40	40	Simultaneous	FQ2	–/12 m	Graftless: elevation and coagulum	1:1 ABG and DPBM
Starch‐Jensen et al. [69]	Parallel	40	40	40	Simultaneous	FQ1	6 m; 18 m/—	Graftless: elevation and coagulum	1:1 ABG and DPBM
Trimmel et al. [8]	Mixed	26	30	53	Delayed	FQ2	–/12 m	A‐PRF + SACBA (3 m healing)	A‐PRF + SACBA (6 m healing)
Torres et al. [70]	Mixed	87 + 5	144 + 10	286	Delayed	FQ2	–/24 m	ABB + PRP(PRGF)	ABB
Triplett et al. [71]	Parallel	160	240	480	Delayed	FQ1/FQ2	6 m/24 m	rhBMP‐2/ACS	ABG
Velasco‐Ortega et al. [72]	Parallel	24	24	44	Simultaneous/delayed	FQ1	9 m/—	T1: β–TCP; T2: β‐TCP + HA 2:1	DBBM
Vincent‐Bugnas et al. [73]	Split‐mouth	8	16	NR	Delayed	FQ2	–/24 m	DBBM + Enamel Matrix derivative	DBBM
Xavier et al. [74]	Parallel	30	30	NR	Delayed	FQ1	6 m/—	FFA	DBBM (1–2 mm)
Zahedpasha et al. [75]	Split‐mouth	10	20	20	Delayed	FQ1	6 m/—	Graftless	Natural Bovine Bone (Cerabone)
Yang and Hwang [76]	Parallel	25	25	NR	Delayed	FQ1	3 m; 24 m/—	rhBMP‐2 + HA	Bovine xenograft
Yu and Qiu [77]	Mixed	19	20	62	Simultaneous	FQ1/FQ2	12 m/12 m	Two bony windows; DBBM (1‐2 mm) + ABG 90:10	One bony window; DBBM (1–2 mm) + ABG 90:10

Abbreviations: β‐TCP, beta‐tricalcium phosphate; ABG, autogenous bone graft; ACS, absorbable collagen sponge; A‐PRF, advanced platelet‐rich fibrin; APBM, anorganic porcine bone mineral; BBGM, biphasic bone graft material; BCP, biphasic calcium phosphate; BMA, bone marrow aspirate; BMAC, bone marrow aspirate concentrate; C, control; CGF, concentrated growth factors; DBBM, demineralized bovine bone matrix (Bio‐Oss); DPBM, deproteinised porcine bone mineral; FFA, fresh frozen allograft; HA, hydroxyapatite; L‐PRF, leukocyte‐and platelet‐rich fibrin; MCA, mineralized cortical allograft; MPM, mineralized plasmatic matrix; NHA, nano‐hydroxyapatite; PRGF, plasma rich in growth factors; PRP, platelet‐rich plasma; rhBMP‐2, recombinant human bone morphogenetic protein‐2; SACBA, serum albumin‐coated bone allograft; T, test; XG, xenograft.

* for FQ1 (focused question 1): months (m) from grafting; for FQ2: months/years (y) from implant loading.

**TABLE 2:** Results of the risk of bias assessment.

Study ID	Randomization process	Deviations from intended interventions	Missing outcome data	Measurement of the outcome	Selection of the reported result	Overall bias
Adali et al. [22]	Low risk	Some concerns	Low risk	Some concerns	Low risk	Some concerns
Aldahouk et al. [23]	Low risk	Some concerns	Low risk	Some concerns	Some concerns	Some concerns
Amam et al. [24]	Some concerns	Some concerns	Low risk	Some concerns	Some concerns	High risk
Attia et al. [25], Schaaf et al. [26, 27]	Low risk	Low risk	Low risk	Low risk	Some concerns	Some concerns
Baldini et al. [28]	Low risk	Low risk	Low risk	Low risk	Low risk	Low risk
Chang et al. [29]	High risk	Some concerns	Low risk	Low risk	Low risk	High risk
Cinar et al. [30]	High risk	Some concerns	Low risk	Low risk	Low risk	High risk
Correia et al. [31, 32, 33]	Low risk	Low risk	Low risk	Low risk	Some concerns	Some concerns
da Silva et al. [34]	Low risk	Some concerns	Low risk	Low risk	Low risk	Some concerns
de Almeida Malzoni et al. [35], Pichotano et al. [36]	Low risk	Low risk	Low risk	Low risk	Low risk	Low risk
Felice et al. [37]	Low risk	Some concerns	Low risk	Low risk	Low risk	Some concerns
Galindo‐Moreno et al. [38]	Low risk	Low risk	Low risk	Low risk	Low risk	Low risk
Galindo‐Moreno et al. [39]	Low risk	Low risk	Low risk	Low risk	Low risk	Low risk
Gorla et al. [40], dos Santos Pereira et al. [41]	Low risk	Some concerns	Low risk	Some concerns	Low risk	Some concerns
Hirota et al. [42]	Low risk	Some concerns	Low risk	Low risk	Low risk	Some concerns
Jia et al. [43]	Low risk	Low risk	Low risk	Some concerns	Low risk	Some concerns
Kaarthikeyan et al. [44]	High risk	High risk	Low risk	Some concerns	Some concerns	High risk
Kamolratanakul et al. [45]	Low risk	Low risk	Low risk	Low risk	Low risk	Low risk
Kandel et al. [46]	Low risk	Some concerns	Low risk	Low risk	Low risk	Some concerns
Kawakami et al. [47]	Low risk	Some concerns	Low risk	Low risk	Low risk	Some concerns
Khaled et al. [48]	Low risk	Some concerns	Low risk	Low risk	Low risk	Some concerns
Kiliç and Güngörmüş [49]	High risk	Some concerns	Low risk	Low risk	Low risk	High risk
Kim et al. [50], Yang and Hwang [76]	Low risk	Low risk	Low risk	Low risk	Low risk	Low risk
Krennmair et al. [51]	Low risk	Low risk	Low risk	Some concerns	Low risk	Some concerns
Kühl et al. [52]	Some concerns	Some concerns	Low risk	Low risk	Low risk	Some concerns
Kühl et al. [53]	Some concerns	Some concerns	Low risk	Low risk	Low risk	Some concerns
Lee et al. [54]	Low risk	Low risk	Low risk	Low risk	Some concerns	Some concerns
Lie et al. [55]	Low risk	Low risk	Low risk	Low risk	Low risk	Low risk
Lindgren et al. [56]	Some concerns	Some concerns	Low risk	Low risk	Low risk	Some concerns
Liu et al. [57]	Low risk	Some concerns	Low risk	Low risk	Some concerns	Some concerns
Meloni et al. [58]	Low risk	Some concerns	Low risk	Low risk	Low risk	Some concerns
Menezes et al. [59]	Some concerns	Some concerns	Low risk	Low risk	Low risk	Some concerns
Nizam et al. [60]	Some concerns	Some concerns	Low risk	Low risk	Low risk	Some concerns
Pang et al. [61]	Some concerns	Low risk	Low risk	Low risk	Low risk	Some concerns
Pereira et al. [62]	Some concerns	Some concerns	Low risk	Low risk	Low risk	Some concerns
Scarano et al. [63]	High risk	Some concerns	Low risk	Low risk	Low risk	High risk
Sedeqi et al. [64]	Low risk	Some concerns	Low risk	Low risk	Low risk	Some concerns
Sokolowski et al. [65]	Some concerns	Some concerns	Low risk	Low risk	Low risk	Some concerns
Stacchi et al. [66]	Low risk	Low risk	Low risk	Low risk	Low risk	Low risk
Starch‐Jensen et al. [67]	Low risk	Low risk	Low risk	Low risk	Low risk	Low risk
Starch‐Jensen et al. [68, 69]	Low risk	Low risk	Low risk	Low risk	Low risk	Low risk
Trimmel et al. [8]	Low risk	Some concerns	Low risk	Low risk	Low risk	Some concerns
Torres et al. [70]	Low risk	Some concerns	Low risk	Low risk	Some concerns	Some concerns
Triplett et al. [71]	Low risk	Some concerns	Low risk	Low risk	Low risk	Some concerns
Velasco‐Ortega et al. [72]	Low risk	Low risk	Low risk	Low risk	Low risk	Low risk
Vincent‐Bugnas et al. [73]	Low risk	Low risk	Low risk	Low risk	Low risk	Low risk
Xavier et al. [74]	Low risk	Low risk	Low risk	Low risk	Low risk	Low risk
Zahedpasha et al. [75]	Some concerns	Some concerns	Low risk	Low risk	Low risk	Some concerns
Yu and Qiu [77]	Low risk	Some concerns	Low risk	Low risk	Low risk	Some concerns

### 3.2 Synthesis of the Results

A summary of the results of the studies included is presented in Table 3. In total, 1756 implants were included from 36 studies. The overall implant survival at one‐year follow‐up is 97% (range: 84.6%–100%). The most documented graft type is xenograft (demineralized bovine bone in 15 studies/637 implants, and porcine bone in two studies/39 implants). Most failures have been reported to occur before loading or in the first 6 months of function.

**TABLE 3:** Main outcomes that are reported in the included studies. It is notable that most of the studies included do not provide clarification about how they avoided/managed unit‐of‐analysis error; for such a reason the reliability of the measurements may be affected in both directions (control or treatment).

Study	FQ1 (graft size) FQ2 (survival/MBL)	Residual bone height, test mean (SD), mm	Residual bone height, control mean (SD), mm	Initial graft height (mm)/volume (cm^3^) test	Graft height/volume change test (gain*)	Initial graft height (mm)/volume (cm^3^) control	Graft height/volume change control (gain*)	Implant survival, test***	Implant survival, control***	MBL mean (SD) test, mm***	MBL mean (SD) control, mm***
Adali et al. [22]	FQ1/FQ2	1–3	1–3	18.7 (1.77)	−6.37 (5.94)%	18.74 (1.61)	−9.32 (8.30)%	100%	100%	NR	NR
Aldahouk et al. [23]	FQ1	3–5	3–5	NR	5.55 (0.93) mm*	NR	2.86 (0.60) mm*	—	—	—	—
Amam et al. [24]	FQ1	3.86 (1.73)	3.55 (2.13)	10.33(3.03) mm	−27.06 (8.1)%	10.31(1.97) mm	−29.54 (20.1)%	—	—	—	—
Attia et al. [25], Schaaf et al. [26, 27]	FQ2	NR	NR	—	—	—	—	93.8%	98.1%	NR	NR
Baldini et al. [28]	FQ1	3.10 (0.93)	3.39 (1.22)	NR	8.71(1.11) mm*	NR	8.5(2.02) mm*	—	—	—	—
Chang et al. [29]	FQ1 FQ2	3.18 (0.77)	3.26 (0.89)	3.95(1.76) mm; 1.77 (0.81) cm^3^	−17.26(12.34)%; −10.22 (4.37)%	4.04(1.57) mm; 1.82 (0.78) cm^3^	−19.10(10.28)%; −12.76 (5.45)%	95.0%	97.6%	< 1.5	< 1.5
Cinar et al. [30]	FQ1	< 5	< 5	2.62 (0.52) cm^3^	−14.41(12.87)%	2.67(0.55) cm^3^	−17.12 (13.55)%	—	—	—	—
Correia et al. [31, 32, 33]	FQ1/FQ2	3.06 (1.13)	3.20 (0.93)	NR	8.71 (2.15) mm*	NR	7.81 (2.34) mm*	92.9%	100%	0.63(0.64)	0.59(0.69)
da Silva et al. [34]	FQ1/FQ2	2.38 (0.75)	3.11 (0.83)	NR	10.62(1.93) mm*	NR	11.56(2.03) mm*	100%	84.6%	NR	NR
de Almeida Malzoni et al. [35], Pichotano et al. [36]	FQ1/FQ2	≤ 4	≤ 4	4mo: 1.69(0.42) cm^3^; 8mo: 1.69(1.05) cm^3^	4mo: 1.1(0.25) cm^3^; −33.13(10.74) %; 8mo: 0.95(0.48) cm^3^; −39.12(16.87) %	1.39(0.53) cm^3^	0.90(0.28) cm^3^; −33.3(13.39)%	100%; 100%	100%	NR	NR
Felice et al. [37]	FQ2	1–3	1–3					95.2%	98.5%	1.01(0.56)	0.93(0.40)
Galindo‐Moreno et al. [38]	FQ1	1.83 (0.89)	1.90 (1.51)	14.42(2.09) mm; 2.29(0.62) cm^3^	Δ −0.61(0.54) cm^3^; (−24.58 (23.6)%)	13.13(2.65) mm; 2.37(0.62) cm^3^	Δ −0.56(0.42) cm^3^; (−24.03 (15.85)%)	—	—	—	—
Galindo‐Moreno et al. [39]	FQ1	2.51 (1.34)	1.73 (0.65)	NR	7.87(4.11) mm*	NR	13.03(3.64) mm*	—	—	—	—
Gorla et al. [40]; dos Santos Pereira et al. [41]	FQ1	< 5	< 5	T1: 1.29(0.94) cm^3^; T2: 0.98(0.50) cm^3^	−38.33(16.64) %; −43.82(18.42) %	1.07(0.48) cm^3^	−45.75 (18.65)%	—	—	—	—
Hirota et al. [42]	FQ1	3.4 (1.1)	3.1 (0.7)	10.6(2.5) mm	Δ −1.4(2.3) mm; (−13.2%)	10(2.8) mm	Δ −1.4(1.2) mm; (−14%)	—	—	—	—
Jia et al. [43]	FQ1/FQ2	3.58 (1.49)	4.12 (1.61)	13.61(1.82) mm	13.32(1.36) mm; (−2.13%)	12.38(1.82) mm	10.92(1.51) mm; (−11.79%)	100%	100%	0.52(0.29)	0.57(0.53)
Kaarthikeyan et al. [44]	FQ1	6.39 (0.81)	5.42 (0.48)	13.67(1.24) mm	11.92(1.39) mm; −12.03(5.08)%	13.51(1.81) mm	10.93(2.53) mm; −19.10(9.36)%	—	—	—	—
Kamolratanakul et al. [45]	FQ2	3.18 (0.10)	3.33 (0.87)	NR	10.44(1.63) mm*	NR	9.46(0.93) mm*	100%	100%	0.86(0.33)m; 1.41(1.01)d	1.69(0.97)m; 1.05(0.78)d
Kandel et al. [46]	FQ1	4.49 (0.76)	4.47 (0.68)	NR	5.4(2.0) mm*	NR	10.2(2.5) mm*	—	—	—	—
Kawakami et al. [47]	FQ1	3.2 (1.1)	3.1 (1.4)	9.8(2.1) mm	Δ −2.0(1.7) mm; 19.4(25.7)%	10.9(1.9) mm	Δ −1.4(2.2) mm; −24.2(22.3)%	—	—	—	—
Khaled et al. [48]	FQ1	4–6	4–6	NR	7.0(0.8) mm*	NR	5.0(1.5) mm*	—	—	—	—
Kiliç and Güngörmüş [49]	FQ1	2.7 (2.57)	4.88 (2.37)	14.77(2.97) mm	Δ −1.58(1.01) mm; (−10.7 (6.8)%)	12.48(2.99) mm	Δ −0.89(0.79) mm; (−7.13 (6.3)%)	—	—	—	—
Kim et al. [50]	FQ1	1.74 (0.72)	2.31 (1.43)	12.67(2.47) mm	12.32(2.16) mm; (−2.76%)	13.22(1.97) mm	14.51(3.27) mm; (+9.76%)	—	—	—	—
Krennmair et al. [51]	FQ1/FQ2	2.9	3	15.3(1.9) mm	6mo: −7.5(5.0)%; 24mo: −12.9(6.7)%	16.2(2.0) mm	6mo: −8.5 (6.3)%; 24mo: −12.4 (5.8)%	100%	100%	0.52(0.19)	0.48(0.15)
Kühl et al. [52]	FQ1	< 3	< 3	2.86(0.77) cm^3^	2.32 (0.57) cm^3^; −18(6.9)%	2.34(0.38) cm3	1.91(0.41) cm^3^; −15.20(6.3)%	—	—	—	—
Kühl et al. [53]	FQ1	< 3	< 3	3.98 cm^3^; 2.76 cm^3^	2.75 cm^3^; −20.45 (13.51)%; 2.33 cm^3^; −16.59 (3.41)%	6.58(3.41) cm^3^; 1.18(0.74) cm^3^	3.41 cm^3^; −15.20(8.37)%; 0.74 cm^3^; −21.50(9.43)%	—	—	—	—
Lee et al. [54]	FQ1	1.83 (0.78)	2.06 (0.43)	13.05(1.25) mm; 1.76(0.72) cm^3^	13.4(1.82) mm; (+2.68%); 1.87(0.77) cm^3^; (+6.25%)	15.06(2.61) mm; 1.74(0.47) cm^3^	15.02(3.17) mm; (−0.26%); 1.90(0.52) cm^3^; (+9.2%)	—	—	—	—
Lie et al. [55]	FQ1/FQ2	4.66 (2.87)	4.53 (2.22)	NR	6.2(4.92) mm*	NR	9.69(4.62) mm*	96.4%	84.6%	NR	NR
Lindgren et al. [56]	FQ2	< 5	< 5	—	—	—	—	95.7%	95.5%	1.32(1.10)	1.10(0.78); [RB 2.1(1.34)]
Liu et al. [57]	FQ1	1.93 (0.77)	2.43 (0.78)	1.40(0.66) cm^3^	1.18(0.4/8) cm^3^; −13.29(8.56)%	2.16(1.29) cm^3^	1.87(1.08) cm^3^; −12.87(5.15)%	—	—	—	—
Meloni et al. [58]	FQ2	1–4	1–4					100%	100%	1.06(0.61)	1.19(0.53)
Menezes et al. [59]	FQ1	NR	NR	G_2_ 0.92(0.42) cm^3^; G_3_ 0.94(0.36) cm^3^; G_4_ 0.90(0.48) cm^3^; G_5_ 1.48(0.90) cm^3^	0.60(0.31) cm^3^; (−34.3 (13.6)%); 0.60(0.23) cm^3^; (−35.8 (20.8)%); 0.48(0.29) cm^3^; (−47.7 (34.6)%); 0.90(0.65) cm^3^; (−39.0 (31.9)%)	G_1_ 1.5(0.42) cm^3^	G_2_ 0.87(0.56) cm^3^; (−41.9 (20.0)%)	—	—	—	—
Nizam et al. [60]	FQ1/FQ2	2.45 (0.79)	2.53 (0.61)	NR	13.6(1.09) mm**	NR	13.53(1.2) mm**	100%	100%	NR	NR
Pang et al. [61]	FQ1	2.92 (2.17)	3.69 (4.85)	14.33(4.85) mm; 1.71(0.30) cm^3^	11.42(6.07) mm; 1.32(0.30) cm^3^; −21.88 (17.33)%	15.68(1.89) mm; 1.91(0.95) cm^3^	11.76(4.48) mm; 1.52(0.68) cm^3^; (−20.5%)	—	—	—	—
Pereira et al. [62]	FQ1	< 5	< 5	T1 0.91(0.47) cm^3^; T2 1.59(0.87) cm^3^	0.47(0.29) cm^3^; −44.2(16.0)%; 1.01(0.59) cm^3^; −37.9(18.9)%	1.07(0.48) cm^3^	0.53(0.22) cm^3^; −45.7(18.5)%	—	—	—	—
Scarano et al. [63]	FQ1	2–3	2–3	3.10(0.32) cm^3^	2.72(4.32) cm^3^; (−12.4%)	2.80(0.22) cm^3^	1.91(0.33) cm^3^; (−31.7%)	—	—	—	—
Sedeqi et al. [64]	FQ1	4.62(1.70)	5.04(1.48)	1.95(1.11) cm^3^	1.59(0.94) cm^3^; −14.87 (16.60)%	2.36(1.24) cm^3^	1.98(1.16) cm^3^; −18.06(9.81)%	—	—	—	—
Sokolowski et al. [65]	FQ1	2.54(1.21)	2.86(0.90)	13.97(1.82) mm	6mo 14.53 (2.38) mm; (−1.41 (3.86)%); 12mo 14.43 (2.28); (−3.76 (3.44)%); 24mo 14.44 (2.30); (−4.26 (3.07)%)	15.53(1.56) mm	15.76(2.36) mm; (−5.46 (2.55)%); 15.60(2.64) mm; (−8.20 (5.56)%); 15.31(2.31) mm; (−10.47 (6.47)%)	—	—	—	—
Stacchi et al. [66]	FQ2	2.03(0.75)	2.03(0.75)	—	—	—	—	96.2%	98.0%	NR	NR
Starch‐Jensen et al. [67]	FQ2	TGI: 4.9(1.4); TGII: 4.6(1.5)	4.7(1.1)	—	—	—	—	100%; 100%	100%	0.58(0.86); 0.23(0.32)	0.23(0.59)
Starch‐Jensen et al. [68]	FQ2	4.9 (0.8)	4.4(0.9)	—	—	—	—	100%	100%	0.45(0.55)	0.50(0.65)
Starch‐Jensen et al. [69]	*FQ1*	*4.9 (0.8)*	*4.4(0.9)*	*0.076(0.017) cm* ^*3*^	*At loading: 0.046(0.009) cm* ^*3*^ *; (−49.01%); 12 m loading 0.039(0.008) cm* ^*3*^ *(−48.88%)*	*0.118(0.024) cm3*	*0.091(0.019) cm* ^*3*^ *; (−22.58%); 0.085(0.016) cm* ^*3*^ *; (−28.18%)*	—	—	—	—
Trimmel et al. [8]	FQ2	2.93(1.14)	3.48(1.04)	—	—	—	—	100%	92.0%	NR	NR
Torres et al. [70]	FQ2	1–7	1–7	—	—	—	—	98.6%	96.2%	NR	NR
Triplett et al. [71]	FQ1/FQ2	< 6	< 6	NR	7.83(3.52) mm*	NR	9.46(4.11) mm*	1y 73.9%; 2y 73.9%	78.5%; 81.5%	0.85 m; 0.95d	0.85 m; 0.95d
Velasco‐Ortega et al. [72]	FQ1	T1 2.5 (0.38); T2 2.63 (0.23)	2.69 (0.46)	NR	T1 12.25(2.33) mm*; T2 11.04(2.29) mm*	NR	11.05(1.53) mm*	—	—	—	—
Vincent‐Bugnas et al. [73]	FQ2	2.6	2.9	12.5(1.2) mm*	NR	12.9(0.7) mm*	NR	100%	100%	NR	NR
Xavier et al. [74]	FQ1	≤ 4	≤ 4	2.48(0.72) cm^3^	1.74(0.82) cm^3^; −31.11 (18.64)%	2.9(0.9) cm^3^	2.56(0.8) cm^3^; −11.6 (4.14)%	—	—	—	—
Zahedpasha et al. [75]	FQ1	4.88 (1.63)	5.36 (1.55)	10.6(1.83) mm	11.08(0.98) mm; (+ 4.5%)	15.43(2.31) mm	14.92(2.47) mm; (−3.3%)	—	—	—	—
*Yang and Hwang* [76]	*FQ1*			*4.11(1.28) cm* ^*3*^	*4.07(1.29) cm* ^*3*^; *−0.97(15.7)%*	*2.95(0.73) cm* ^*3*^	*2.83(0.78) cm* ^*3*^; *−4.2(7.4)%*	—	—	—	—
Yu and Qiu [77]	FQ1/FQ2	2.50 (0.39)	2.35 (0.36)	NR	11.94(1.71) mm**	NR	12.19(1.25) mm**	100%	100%	0.55(0.6)	0.4(0.71)

Abbreviations: d, distal; FQ1, focused question 1; FQ2, focused question 2; m, mesial; MBL, marginal bone loss; mo, months; NR, not reported; RB, residual bone; SD, standard deviation; y, year. The two lines in italics [69, 76] represent multiple articles of a single study.

* Gain (graft height).

** Graft + ridge height.

*** Where not specified, the longest follow‐up is considered.

Tables 4 and 5 summarize the graft reduction in volume and height, respectively, after 6 months of the sinus augmentation procedure, expressed as a percentage respect to baseline, and divided according to the graft type. The graft material showing the highest volume resorption rate is autogenous bone alone (four studies, 44 sinuses), while xenografts (12 studies, 180 sinuses) showed the lowest reduction. Fewer data were available for graft height changes (Table 4).

**TABLE 4:** Summary of graft volume change percentage according to the material type after 6 months. Only materials in which at least two studies were found are included.

Graft type	Number of studies	Number of sinuses treated	Weighted mean (%)	Range (%)
AGF + any graft	3	34	29.74	14.4–39.1
Alloplastic bone	6	59	34.73	15.2–47.7
Autograft + alloplast	6	55	35.02	18.0–39.0
Autograft + xenograft	4	32	22.14	16.6–24.6
Autograft	4	44	45.02	41.9–45.8
Xenograft	12	180	15.68	4.2–36.7

Abbreviation: AGF, autologous growth factor.

**TABLE 5:** Summary of graft height change percentage according to the material type after 6 months. Only materials in which at least two studies were found are included.

Graft type	Number of studies	Number of sinuses treated	Weighted mean	Range
AGF + any graft	5	46	15.95%	6.37–29.54%
Alloplastic bone	2	32	3.44%	1.41–5.46%
Xenograft	6	97	16.51%	7.5–24.2%

Abbreviation: AGF, autologous growth factor.

Due to the heterogeneity among the studies regarding the surgical protocols, the grafting materials, the outcome measures, the follow‐up periods, and the methods of measurement of the outcomes, no pairwise meta‐analysis was performed, and the GRADE framework was not used. The findings from the studies included are presented in a descriptive manner, considering different techniques and grafting materials.

#### 3.2.1 Graftless SFA

In six RCTs (seven papers) [23, 44, 48, 55, 63, 68, 69, 75], graftless SFA was studied. In three studies, graftless SFA was compared to deproteinized bovine bone (DBBM) [55, 68, 69, 75], and in two of them, DBBM was associated with autogenous bone graft (ABG) [55, 68]. In one study, graftless SFA was compared to collagenated PB [63], in one to hydroxyapatite nanoparticles [48], and in one to PRF [44]. In three studies, a collagen membrane was positioned to cover the lateral osteotomy) [63, 68, 69, 75]; in one study, a Ti‐reinforced membrane was used [44], and in one study, a heterologous cortical bone lamina was used in one arm [63].

The study by Aldahouk [23], comparing two graftless technique with different size of the antrostomy, found that when the antrostomy is small (5 mm), a significantly higher vertical bone gain is obtained compared to large antrostomy (10 mm), being the mean bone gain 5.55 ± 0.93 and 2.86 ± 0.60 mm, respectively. The study by Kaarthikeyan [44] compared a graftless technique and the use of PRF, revealing no significant differences in terms of bone gain after 12 months. Khaled et al. [48] found that the use of HA nanoparticles may lead to a significantly higher bone gain than sites treated graftless (7 ± 0.8 versus 5 ± 1.5 mm) after 6 months. When compared to sites treated with a mixture of DBBM and autogenous bone, graftless sites demonstrated a lower amount of bone gain in two studies [55, 68, 69] that evaluated the outcomes after 4, 6, and 12 months. In one study, DBBM was used alone in the test group [75], leading to significantly better results in terms of new bone volume as compared to graftless group. Similar results were found when porcine bone was tested in comparison to no graft [63]. In one study, the number of failures in graftless group (one out of 29) was lower than the number of failures in DBBM + autogenous bone group (four out of 30) after a follow‐up of 57–88 months [55]. The study by Starch‐Jensen and coworkers revealed no significant differences between graftless group and DBBM + autogenous bone group regarding marginal bone loss after one year [68, 69]. None of the studies included in this group reported on PROMs.

#### 3.2.2 Xenografts

##### 3.2.2.1 Bovine Bone

DBBM was used in a total of 28 studies (31 articles) [28, 29, 34, 35, 36, 37, 38, 39, 43, 45, 46, 50, 51, 53, 54, 55, 56, 57, 60, 61, 64, 66, 68, 69, 70, 72, 73, 74, 75, 76, 77]. In one study, a DBBM group was compared to fresh frozen allograft [74]; in one study, it was compared to both β‐TCP and β‐TCP + HA (2:1); in two studies, different brands of DBBM were compared [34, 61]; in two studies, in both groups, the same bone graft was used but with different types of access window [28, 77]; in one study, the same DBBM was used but in one group, the bone window was repositioned in place [43]; in one study, the same DBBM was used but the healing period was 5 months in one group and 8 months in the other [57]; one study compared different particle sizes (1–2 vs. 0.25–1 mm) [45]; one study compared different treatment protocols, namely SFA with simultaneous implant placement vs. SFA and delayed (4 months) implant placement [37]. One study compared DBBM and DBBM + cortical allograft [64]; in one study, DBBM + ABG (80:20) was compared to porcine bone and ABG (80:20) [38]; in three studies, DBBM was compared to DBBM and L‐PRF [35, 36, 60]; in one study, DBBM was compared to DBBM and PRP [70]; in one study, in the test group, DBBM was associated with enamel matrix derivative [73]; in one study, DBBM and bone marrow aspirates from different skeletal regions were compared [53]; in two studies, DBBM was compared to porcine bone alone [51, 54]; in one study, DBBM and ABG (80:20) were compared to a group treated with biphasic phycogenic biomaterial [39]; in one study, DBBM was compared to gelatine sponge [46]; in one study, gelatin sponge and DBBM were compared to DBBM alone [29]; in one study (two articles) DBBM was compared to recombinant human Bone Morphogenetic Protein‐2 (rhBMP‐2) and hydroxyapatite [76, 78]; in three studies, DBBM was compared to graftless procedure [55, 68, 69, 75]; in two studies, DBBM was compared to b‐TCP + HA (2:1) [56, 73]; in one study, DBBM was compared to sintered nanohydroxyapatite [66].

Some of the included studies reported a significant difference in the outcomes between the test and the control group. Baldini et al. [28] found that the smaller the bone access, the less the impact on patients' quality of life after the intervention, without any effect on clinical and radiographic outcomes. The study by Kamolratanakul et al. found that when larger DBBM particles were used, it was possible to obtain a higher bone volume than when the graft was composed of smaller particles [45]. The study by Yu and Qiu [77] found better outcomes if two small bone windows were done as compared to just one bigger in terms of clinical and radiographic results. Jia et al. [43] reported a clinical and radiographic advantage in repositioning the bone window in place after grafting. As compared to other biomaterials, DBBM performed better than allograft, with a lower resorption of the graft, in one study [74], better than phycogenic material in one study [39], and better than gelatin sponge in one study [46]. The addition of rhBMP‐2 to HA material resulted in significantly better results than DBBM alone, as reported in one study [76]. As reported in two studies, described in the previous section, graftless solutions performed worse than DBBM in terms of bone gain [55, 68, 69].

##### 3.2.2.2 Porcine Bone

Porcine bone (PB) was used alone in seven studies (nine articles) [31, 32, 33, 38, 42, 47, 51, 54, 63, 67]. In two studies, PB was used in both the control and test groups, but in different conditions, namely placing a collagen membrane subjacent to the sinus mucosa in the test group and not in the control groups [42] and performing two different types of antrostomy [47]. In one study, PB was compared to ABG [31, 32, 33], while in one study, a combination of PB and ABG was compared to DBBM and ABG [38], and in one study, PB and ABG were compared to ABG alone [67]. In two studies, PB was compared to DBBM alone [51, 54], and in one study, PB was compared to MSFA without graft [63].

Two studies reported evidence of a statistical difference between the study groups [47, 63]. The results of Scarano's research were described earlier. In the study by Kawakami et al., the researchers examined the data about 10 subjects with a follow‐up of 9 months [47]. The authors found that the more cranial the antrostomy was, the higher the amount of bone augmentation they could find.

#### 3.2.3 Allograft

Allograft was used, in different conditions, in four studies [8, 22, 64, 74]. In one study, fresh frozen cortico‐cancellous bone blocks from distal epiphyses (milled during the surgical procedure) were used [74], while in the other studies, allograft was in granules.

In two studies, allograft was associated with platelet growth factors (concentrated growth factors, CGF [22] and A‐PRF [8]). In one study, allograft was combined with DBBM and compared to DBBM alone [64]. In one study, the graft was compared to DBBM alone [74].

In the study by Trimmel and coworkers, two healing periods were evaluated, reporting no significant differences between the 3‐ and 6‐month healing periods when allograft was combined with A‐PRF [8]. One study reported the absence of a significant difference between allograft and DBBM in terms of clinical results at a 6‐month follow‐up [74]. Also, the study by Sedeqi reported no evidence of a difference between the two groups [64]. The study by Adali and coworkers found evidence of a difference in terms of bone height 6 months after surgery, favoring the group with adjunctive CGF [22].

#### 3.2.4 Alloplastic Bone

##### 3.2.4.1 Beta Tricalcium Phosphate

A total of 11 studies (12 articles) evaluated the use of β‐TCP as a bone substitute material in MSFA [24, 30, 40, 41, 50, 52, 56, 59, 65, 67, 72, 79]. In five studies, β‐TCP was used in association with HA (biphasic calcium phosphate) [50, 52, 56, 65, 72]; in three studies, it was associated with growth factors (A‐PRF [24], PRF [30], PRP [79]); in three studies, it was used with ABG [40, 41, 59]. In one study, β‐TCP was used associated with both HA and autogenous bone [52].

In one study, β‐TCP and A‐PRF were compared to calcium sulfate and A‐PRF without any significant difference in bone gain between the two groups [24]. In two studies, TCP and platelet concentrates were compared to β‐TCP alone without finding, in both, any evidence of a significant difference in bone resorption [30, 79]. In three studies, a combination of β‐TCP and autogenous bone was compared to autogenous bone alone [40, 41, 59]. In one study, a combination of β‐TCP, HA, and ABG was compared to ABG alone [67]. In two studies, a combination of β‐TCP and HA was compared to DBBM without any significant difference between the two groups for the outcomes considered [56, 72]. The study by Kuhl et al. demonstrated that β‐TCP + HA and adjunctive (1:1) ABG resorbed similarly to β‐TCP + HA alone [52]. When compared to HA alone, β‐TCP + HA demonstrated a higher stability of the bone volume over time, although no differences in terms of implant biological and technical complications were found [65]. In the study by Kim and coworkers, β‐TCP was associated both to HA and rhBMP‐2 without any significant difference between groups [50].

##### 3.2.4.2 Hydroxyapatite

Five studies (six articles) compared the use of synthetic HA in MSFA to other bone substitute material [48, 50, 52, 65, 67, 76]. In one study, β‐TCP and HA were compared to almost pure HA [65]; in one study, HA was compared to graftless MSFA intervention [48]; in one study, β‐TCP + HA and HA with adjunctive rhBMP‐2 were compared to DBBM [50, 76]. In one study, β‐TCP, HA, and ABG were compared to β‐TCP + HA [52]. In one study, HA, with β‐TCP and ABG, was compared to ABG alone [67]. As previously reported, HA performed significantly better than graftless MSFA [48], while other studies did not report any evidence of a significant difference considering the clinical outcomes.

#### 3.2.5 Autogenous Bone

ABG was used in 14 studies (20 articles) included in the review [25, 26, 27, 31, 32, 33, 38, 39, 40, 41, 52, 55, 58, 59, 62, 67, 68, 69, 71, 77]. In several studies, ABG was used combined with other bone substitute materials, such as DBBM [38, 39, 55, 58, 68, 69, 77], β‐TCP and/or HA [40, 41, 52, 59, 67], PB [31, 32, 33, 38, 67], bioactive glass [59, 62], and biphasic phycogenic biomaterial [39]. ABG was associated with growth factors in two studies [25, 26, 27] and with rhBMP‐2 in one study [71].

For the outcomes considered, two studies reported the evidence of a significant difference between the test and control group [39, 55], one reporting worse results for biphasic phycogenic material [39] and the other reporting a lower increase in available bone in the group treated without graft [55].

#### 3.2.6 Autologous Growth Factors/Platelet Concentrates

Autogenous growth factors (GFs) were evaluated in 11 studies (13 articles) [8, 22, 24, 25, 26, 27, 30, 35, 36, 44, 49, 60, 70]. Different GFs were studied: CGF [22], PRF (A‐PRF or L‐PRF) [8, 24, 26, 27, 30, 35, 36, 44, 60], PRP [25, 26, 27, 49], and PRGF [70]. In one study, GFs were used alone, without the association with a bone substitute material [44]. In two studies, GFs were used with allografts [8, 22]; in three studies, they were used with β‐TCP [24, 30, 49]; in one study with calcium sulfate [24]; in one study with ABG [25, 26, 27]; and in four studies, they were used with DBBM [35, 36, 60, 70].

For the outcomes considered in both the focused questions, only one of the studies dealing with the use of GFs found evidence of a significant difference between groups, favoring the group in which CGF was used [22].

#### 3.2.7 Bone Morphogenetic Proteins

Two studies (3 papers) evaluated the use of rhBMP‐2, associated with other materials [50, 71, 76]. In one study, rhBMP‐2 is used with HA and compared to DBBM [50, 76], while in another study, it is used with absorbable collagen sponge and compared to ABG. None of the studies found evidence of a significant effect of adjunctive rhBMP‐2 for the considered outcomes, as compared to control groups.

#### 3.2.8 Other Biomaterials

Five studies evaluated materials different from those considered above [24, 39, 59, 62, 73]. In one study, b‐TCP and PRF were compared to calcium sulfate and PRF [80]. Bioactive glass was compared to ABG in two studies [59, 62]. HA extracted from algae and mixed with ABG was compared to a combination of DBBM and ABG in one study [39]. In one study, enamel matrix derivative was mixed with DBBM and compared to DBBM alone [73].

None of the studies reported the presence of a statistically significant difference between the study groups.

#### 3.2.9 Use of a Membrane to Cover the Antrostomy

In three studies, different membranes were used in the control and test group [24, 51, 63]. In one study, a resorbable collagen membrane was placed to cover the antrostomy in one group, while in the other group it was not used [24]. In one study, two different types of resorbable collagen membrane were evaluated [51]. Then, in one study, a resorbable membrane was used in one group, and in the other group, the antrostomy was covered with a cortical lamina [63]. None of the studies reported a significant effect on the considered outcomes of the membrane used to cover the antrostomy.

Only a small proportion of the studies included reported data on marginal bone loss over the years, as can be seen in Table 3. Therefore, no quantitative analysis on such outcome was performed.

## 4 Discussion

The present systematic review of the literature included randomized controlled trials about clinical, radiographic, and patient‐reported outcomes after lateral MSFE with different techniques and biomaterials. Despite not being possible to perform a pairwise meta‐analysis, due to the heterogeneity of the studies, the lack of individual participant data, and the presence of a unit‐of‐analysis errors that affected most of the included studies, the systematic review allowed us, in the opinion of the authors, to respond to the focused questions we posed. Specifically, we found no evidence that a biomaterial or a combination of them may perform better than others considering the graft size and stability after intervention, and implant‐related outcomes. We found that MSFA without using any biomaterial caused a significantly lower bone augmentation as compared to MSFA with bone grafts, although such a difference seems not to imply the impossibility of implant placement and successful rehabilitation. From these findings, we may hypothesize that the choice of grafting material and the size of augmentation do not have a significant effect on the achievement of the goal of MSFE, which is the safe placement of dental implants in the posterior maxilla for rehabilitation of masticatory function. The choice of grafting material may be dictated by reasons other than clinical effectiveness. For example, some studies investigated the patients' preferences based on ethical, cultural, psychological, or religious reasons. Grafts from animals or human cadavers or synthetic grafts may represent a concern for some people, leading to the rejection of the use of certain bone graft types in their treatments [81, 82, 83]. Such motivations are not of secondary importance and seem to be undervalued in clinical trials involving the placement of bone grafts [84]. Another aspect that can influence the graft choice is the cost‐effectiveness, whose consideration is part of the health technology assessment process.

It would be important to assess the stability of different grafting materials in the long term, to evaluate if different resorption rates are associated with different implant survival rates. Unfortunately, no such data were available in the included studies. Very few studies in fact assessed graft stability in a follow‐up longer than six months [24, 43, 44, 47, 65, 72, 76, 77]. The effect of a covering membrane on graft height or volume stability might be negligible on the outcomes considered, based on the available evidence.

Even though MSFA with lateral approach is one of the most popular treatments for rehabilitating atrophic maxilla, several alternatives have been proposed over the years, such as tilted implants, short implants, distal cantilevers, and transcrestal sinus floor augmentation, while zygomatic implants and pterygoid implants can be considered for the most severe maxillary atrophies [3]. Most of these techniques were designed to exploit the residual alveolar bone, avoiding demanding grafting procedures that may cause discomfort to some patients. The choice among the different treatment options depends on different factors and conditions, among which are clinician's skills and patient preferences [3]. In terms of clinical success, MSFA and standard‐length implants have demonstrated, as compared to short implants without any augmentation procedure, similar outcomes, as reported in several systematic reviews of the literature on RCTs [14, 85, 86, 87]. Similarly, following the respective indications, the other alternatives to MSFA performed well in terms of clinical and patient‐reported outcomes [3, 88, 89]. Indeed, in the systematic review published in 2019, Raghoebar and coworkers, by evaluating the long‐term (more than five years) outcomes of the MSFA procedure, through a meta‐analysis, reported a weighted annual rate of implant loss of 0.43% with low complications [15]. These data confirmed what was found in another study that did not find any difference in terms of prosthesis survival between sites treated with different biomaterials [90].

Focusing on comparative studies addressing the efficacy of different treatment strategies and biomaterials, considering histomorphometric results, the scientific literature reported that the evidence of a difference among different approaches is lacking [6, 18, 91]. The systematic review published by our group in 2016, which examined the histomorphometric results after MSFA by using various biomaterials, found that good results in terms of new bone formation may be obtained through different strategies with several biomaterials, notwithstanding ABG resulted in a slightly higher amount of new bone as compared to xenografts or synthetic materials [6]. A network meta‐analysis based on histomorphometric data from lateral MSFA procedures found that the adjunct of autologous GFs had the best effect among all grafting materials in a follow‐up shorter than 6 months, while for longer follow‐ups, a mixture of xenograft and allograft produced the best outcome [92]. A recent network meta‐analysis that also considered the effect of residual bone height (RBH) reported more favorable results in terms of new bone formation for xenografts in combination with allografts or alloplastic materials when RBH is < 4 mm, while for RBH > 4 mm autogenous bone alone or in combination with xenograft yielded the best outcomes [91].

The merit of the present systematic review of the literature stands in the fact that, to our knowledge, it is a comprehensive appraisal of the existing literature about randomized comparative studies reporting clinical outcomes on different strategies for MSFA with a lateral approach. A recent systematic review addressing the same topic included only five studies, without the possibility of performing a meta‐analysis, and reported a substantial equivalence in outcomes using one grafting material or another [93]. Compared to that review, which aimed at investigating bone‐implant contact in histomorphometric analysis of harvested implants, the results of the present investigation were referred to a much higher number of papers, investigating not just bone grafting materials but also bioactive factors and combinations of them.

The main limitations of the present study are (a) the impossibility to perform a meta‐analysis of comparative studies; (b) the wide heterogeneity in the techniques used to assess the amount of bone augmentation and graft stability over time (graft volume or graft height changes; inclusion or exclusion of the residual ridge in the height measurement; the exact location of measurement; different settings of the CBCT device; data provided as mean values with standard deviation, mean difference, percentage changes, or median and confidence intervals); (c) the lack of data about patient‐reported outcomes (PROMs); (d) unit‐of‐analysis errors in the included studies that prevented the possibility of performing a quantitative synthesis; and (e) the short follow‐up for implant survival rate (13 of the 22 studies reporting on FQ2 had a follow‐up of 12 months, and only two studies had a follow‐up longer than 36 months). Indeed, a previous systematic review on long‐term implant survival in MSFA reported that the incidence of late failures (occurring later than three years) is an extremely rare event, equal to about 0.4%, with 80% of failures occurring within the first year of function [94]. The authors observed a substantial presence of unit‐of‐analysis error in the included studies, which is a typical issue in cluster randomized studies. Such error may produce inflated/erroneous conclusions, also by invalidating *p* values in tests comparing means and other issues related to the amplification of one result over another.

## 5 Conclusion

Considering the exposed limitations, according to the findings of the present review that derive from the qualitative synthesis of the results of included studies, it can be hypothesized that the success of the MSFA procedure may not depend on the graft type, at least in the short term, also considering that implants can be successfully placed and loaded using graftless protocols. The graft stability showed some differences among graft types after six months of placement, but this apparently did not affect implant survival. Data on long‐term graft dimensional stability from evidence‐based studies are still lacking. The choice of the grafting material may not depend on the efficacy of a given product over the others but can rely upon patient and clinician preferences as well as economic, ethical, or religious considerations. Clinicians must carefully evaluate patient‐specific factors, such as bone quality, quantity, and medical history, to select the most appropriate technique and material for optimal outcomes in sinus augmentation procedures. It is suggested that future studies (that should consider PROMs and very standardized outcome measures) will take into account a solid methodology in randomization of the subjects, by following criteria for analysis that can address the issues related to unit‐of‐analysis considerations. The scientific knowledge in this particular field would benefit from more well‐designed RCTs with longer follow‐up.

## Author Contributions

Concept/design: M.D.F. and S.C. Search strategy: S.C. and M.D.F. Screening/data collection: M.D.F., S.T., and S.C. Data analysis/interpretation: S.C. and M.D.F. Manuscript draft: S.C. and M.D.F. Critical revision of manuscript: S.T. and M.D.F. All authors contributed and approved equally.

## Conflicts of Interest

The authors declare no conflicts of interest.

## Supporting information


**Appendix 1** **Search strategy**
Appendix 2. List of the excluded studies (*n* = 67), with the main reason for exclusion.

## Data Availability

The data that support the findings of this study are available from the corresponding author upon reasonable request.
