# Supplementary material for: Efficacy of Different Materials for Maxillary Sinus Floor Augmentation With Lateral Approach. A Systematic Review
Source: Clin Implant Dent Relat Res. 2025 May 22;27(3):e70053. doi: 10.1111/cid.70053 (PMC12099286; doi:10.1111/cid.70053)
Supplement: Supplementary file 1 — Appendix 1 Search strategy Appendix 2. List of the excluded studies (n = 67), with the main reason for exclusion. [file CID-27-0-s001.docx]

**Appendix 1**

**Search strategy**

**MEDLINE via OVID (29^th^ May 2024): 920**

|  | **MeSH terms** | **Free-text search** |
| --- | --- | --- |
| ***Intervention*** | Exp Maxillary sinus  AND  Surgery, Oral | “Maxillary sinus”  AND  (“floor elevation” OR augmentation* OR elevation* OR “floor augmentation” OR lift OR “volume augmentation” OR increase OR graft*) |

By using the tools provided by the interface, we included only studies on humans, and we excluded systematic reviews of the literature *a priori*.

**Cochrane library via CENTRAL (29^th^ May 2024): 727**

|  | **MeSH terms** | **Free-text search** |
| --- | --- | --- |
| ***Intervention*** | Exp Maxillary sinus  AND  Surgery, Oral | (“Maxillary sinus” OR Sinus)  AND  (“floor elevation” OR augmentation* OR elevation* OR “floor augmentation” OR lift OR “volume augmentation” OR increase OR graft*) |

**EMBASE (29^th^ May 2024): 2953**

|  | **EMTREE terms** | **Free-text search** |
| --- | --- | --- |
| ***Intervention*** | ‘maxillary sinus’/exp  AND  (‘oral surgery’/exp OR ‘bone graft’/exp OR ‘bone augmentation’)  OR  (‘maxillary sinus floor augmentation’ OR ‘maxillary sinus floor elevation’) | “Maxillary sinus”  AND  (“floor elevation” OR augmentation* OR elevation* OR “floor augmentation” OR lift OR “volume augmentation” OR increase OR graft*) |

[humans]/lim AND [clinical study]/lim

3922 after removal of duplicates

Appendix 2. List of the excluded studies (n=67), with the main reasonfor exclusion.

| **Study** | **Reason for exclusion** |
| --- | --- |
| Ahmed et al. 2017 | Not an RCT |
| Albadani et al. 2024 | Not an RCT |
| Altintas et al. 2013 | No data relevant for the review |
| Bacevic et al. 2021 | Only one group treated with lateral sinus floor elevation |
| Bae et al. 2010 | Not an RCT |
| Bahaa-Eldin et al. 2017 | Insufficient sample size |
| Bonardi et al. 2017 | Only histomorphometric evaluation |
| Bonardi et al. 2023 | Only histomorphometric evaluation |
| Boos Lima et al. 2017 | Insufficient data provided |
| Chackartchi et al. 2011 | Only histomorphometric and micro-CT evaluation |
| Chaushu et al. 2020 | Not an RCT (the word “random” is only in the abstract) |
| Cordioli et al. 2001 | Not an RCT |
| Corinaldesi et al. 2013 | Follow-up too short (4 months for graft size change) |
| Danesh-Sani et al. 2016 | Only histomorphometric evaluation |
| de Almeida Barros Mourao et al. 2019 | Data of test and control are not split |
| Del Fabbro et al. 2013 | No data relevant for the review (only post-operative quality of life) |
| Deluiz et al. 2017 | The same graft was used in test and control, with different healing times (4 and 6 months) |
| Deng et al. 2022 | Only one group treated with lateral sinus floor elevation |
| dos Anjos et al. 2016 | No data relevant for the review (only implant stability quotient was assessed) |
| Dursun et al. 2016 | Only histomorphometric and micro-CT evaluation |
| Dursun et al. 2015 | Only histomorphometric and micro-CT evaluation |
| Flichy-Fernandez et al. 2019 | No data relevant for the review |
| Fouad et al. 2018 | No data relevant for the review |
| Galindo-Moreno et al. 2008 | Not an RCT |
| Galindo-Moreno et al. 2018 | Not an RCT |
| Galindo-Moreno et al. 2011 | No data relevant for the review |
| Gouda et al. 2018 | Insufficient sample size |
| Harlos et al. 2022 | Only histomorphometric evaluation |
| Hermund et al. 2012 | Only histomorphometric evaluation |
| Jonasson et al. 2017 | Not a clinical study (rat model) |
| Jiang et al. 2023 | Not a follow-up study |
| Kaigler et al. 2015 | Follow-up too short (6 months for implant survival) |
| Karagah et al. 2022 | No data relevant for the review (only implant stability quotient was assessed) |
| Kim et al. 2015 | Only histomorphometric evaluation |
| Kim & Hong 2010 | Only histomorphometric evaluation and low sample size |
| Kim et al. 2009 | Not an RCT |
| Klein et al. 2019 | Not an RCT |
| Kohal et al. 2015 | Only histomorphometric evaluation |
| Kolerman et al. 2019 | Only histomorphometric evaluation |
| Kolerman et al. 2017 | Only histomorphometric evaluation |
| Kotsu et al. 2022 | Only histomorphometric evaluation |
| Kühl et al. 2013 | Only micro-CT analysis |
| Kumar et al. 2015 | Not an RCT |
| La Monaca et al. 2018 | Only histomorphometric evaluation |
| Liu et al. 2023 | Not an RCT |
| Nartins et al. 2021 | No data relevant for the review (only post-operative quality of life) |
| Merli et al. 2022 | Not an RCT |
| Molnar et al. 2022 | Only histomorphometric evaluation and other parameters not relevant for the review |
| Mordenfeld et al. 2013 | Lateral ridge augmentation, not sinus elevation |
| Pereira et al. 2021 | Multiple publication (Menezes et al. 2021), reporting data not relevant for this review |
| Pichotano et al. 2018 | Not an RCT |
| Rickert et al. 2014 | Data of test and control are not split |
| Rickert et al. 2011 | Only histomorphometric evaluation |
| Rodriguez y Baena et al. 2013 | No data relevant for the review (missing pre- and post-operative CBCT) |
| Schmitt et al. 2013 | Only histomorphometric evaluation |
| Schulze-Späte et al. 2016 | No data relevant for the review |
| Schwartz et al. 2008 | Not an RCT |
| Shiezadeh et al. 2023 | No data relevant for the review |
| Shirmohammadi et al. 2014 | Follow-up too short (5 months) and data not relevant for the review |
| Starch-Jensen et al. 2021 | No data relevant for the review (only PROMs at 1 week and 1 month) |
| Starch-Jensen et al. 2022 | No data relevant for the review (only PROMs at 1 week and 1 month) |
| Stavropoulos et al. 2011 | Only histomorphometric evaluation |
| Stavropoulos et al. 2007 | Only transcrestal augmentation not lateral technique |
| Szabo et al. 2005 | Only histomorphometric evaluation |
| Torres García-Denche et al. 2013 | Data not usable for the review (doesn’t report implants per group, only sinus-based analysis) |
| Trimnh et al. 2019 | Only transcrestal augmentation not lateral technique |
| Whitt et al. 2020 | Follow-up too short (4 months for graft size change), and incomplete data |

CBCT: cone-beam computed tomography; CT: computed tomography; PROMS: patient-reported outcome measures; RCT: randomized clinical trial
